# First person – Nefeli Skoufou-Papoutsaki

**DOI:** 10.1242/dmm.050518

**Published:** 2023-10-05

## Abstract

First Person is a series of interviews with the first authors of a selection of papers published in Disease Models & Mechanisms, helping researchers promote themselves alongside their papers. Nefeli Skoufou-Papoutsaki is first author on ‘ Efficient genetic editing of human intestinal organoids using ribonucleoprotein-based CRISPR’, published in DMM. Nefeli is a postdoc in the lab of Douglas J. Winton at the CRUK Cambridge Institute, Cambridge, UK, investigating early cancer and stem cell research.


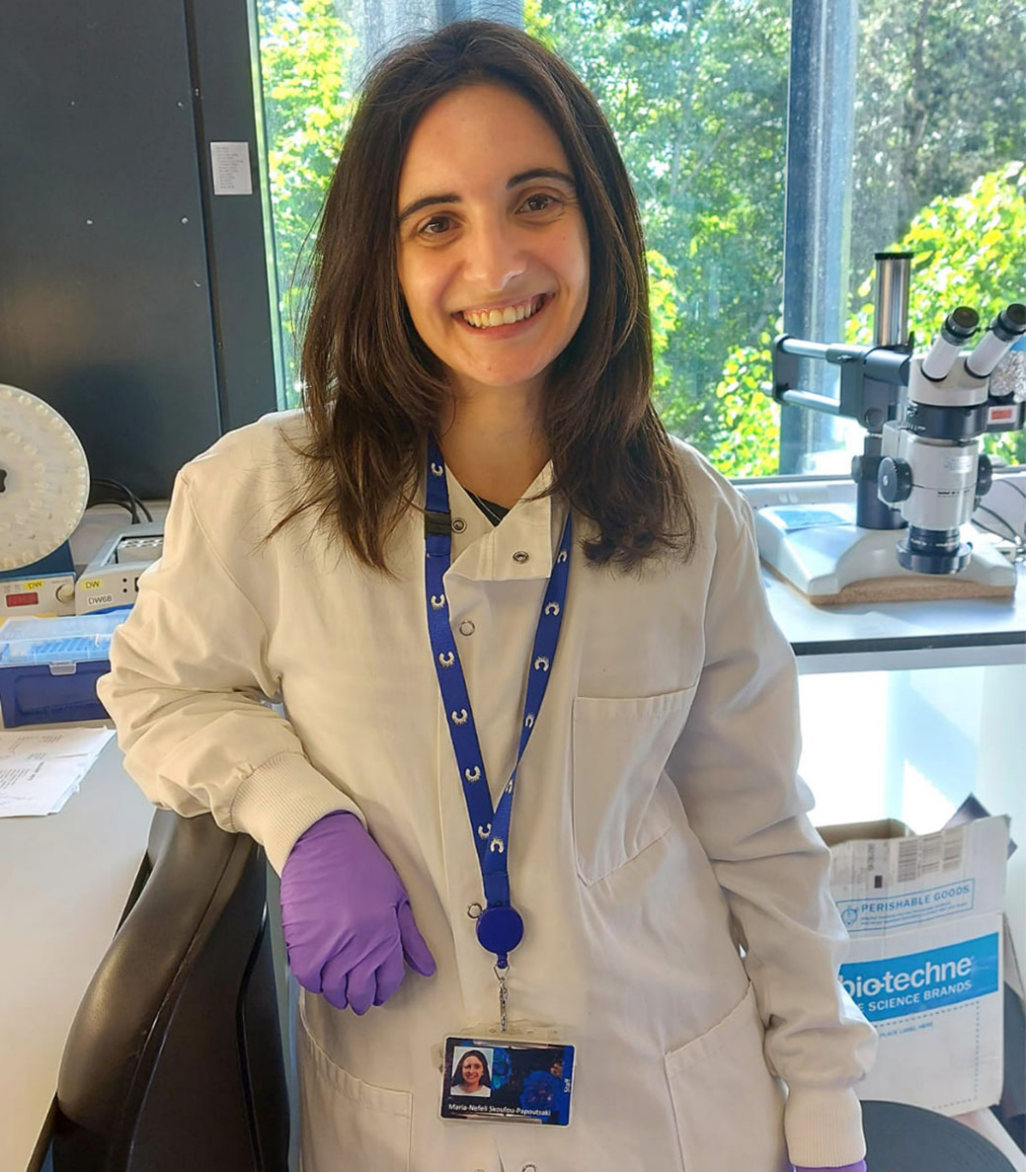


**Nefeli Skoufou-Papoutsaki**

To model human disease, scientists have recently started using patient samples to generate mini-organs (organoids) that have similar functions and characteristics to the organ found in the patient […]

**How would you explain the main findings of your paper to non-scientific family and friends?**

To model human disease, scientists have recently started using patient samples to generate mini-organs (organoids) that have similar functions and characteristics to the organ found in the patient, and contain all the relevant cell types. Errors (mutations) in our DNA are important causative factors of diseases, such as cancer. If these mini-organs can be edited at the DNA level to contain these errors, we could understand the effect they have on disease development, progression or treatment. However, so far, genetic editing of organoids has been technically challenging. We developed a method to very efficiently and accurately introduce errors in the DNA of mini-guts that can allow us to model colorectal cancer in a dish in a quick and simple way. These modified small organ-like structures could help us answer simple cause and effect questions about disease biology and give us a better representation of a patient's condition compared with using animals or human cells.

**What are the potential implications of these results for your field of research?**

Our method for CRISPR-editing organoids is three times more efficient than previously published methods (up to 98% gene knockout), without bio-hazard issues and a turnaround time of ∼1 month from start to finish compared with, for example, lentiviral methods that can take up to 2–3 months. This means that earlier passage organoids can be used, which more closely resemble the patient's characteristics and allow more time for additional experiments, opening up more options for disease modelling and treatment testing. We are hoping that our principle and approach will not only apply to human intestinal organoids but, with simple optimisations of some conditions, could be used for editing organoids from other tissues, too.

However, [patient-derived organoids] offer an epithelial-centric view of the disease […] and co-cultures with mesenchymal or immune cell populations […] have so far proven to be rather challenging.

**What are the main advantages and drawbacks of the experimental system you have used as it relates to the disease you are investigating?**

Patient-derived organoids have the obvious advantage of being a more translational model than human cell lines that better recapitulate the complexity of the organ of interest. In addition, they could be used to reduce the use of animal models with their associated ethical concerns. However, they offer an epithelial-centric view of the disease (colorectal cancer in our case) and co-cultures with mesenchymal or immune cell populations, to make a more accurate model, have so far proven to be rather challenging. This makes organoids, perhaps, a too simplistic model for some specific questions. In addition, some questions, such as tumour initiation or progression, cannot be addressed without the need for a more functional *in vivo* model. Finally, they cannot be grown indefinitely and are, therefore, time sensitive. Overall, while organoids could be ideal to answer some cause-and-effect questions, and could be used for some more translational research, such as pre-clinical drug testing, ideally, they should be used in combination with *in vivo* models for a more holistic understanding of the biological complexity.

**Figure:**
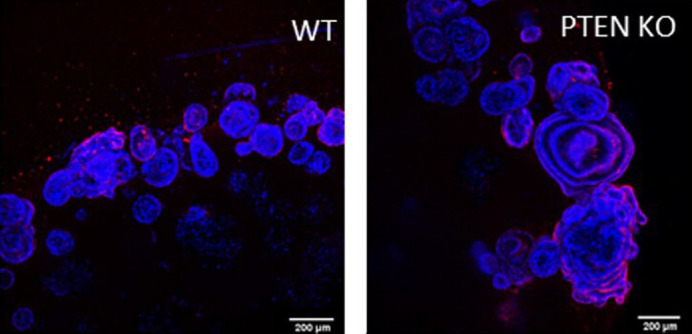
**Confocal images of human colonic organoids generated using the described CRISPR-Cas9 ribonucleoprotein-based method for genome editing.** Organoids with engineered nonsense mutations in the PTEN tumour suppressor gene (PTEN KO) and isogenic controls (WT) are shown. Loss of PTEN leads to phenotypically larger organoids containing more cells, indicative of enhanced proliferation. The proliferation marker MCM2 is shown in red, nuclei are stained with DAPI (blue).

**What has surprised you the most while conducting your research?**

I think the most surprising result we got was the level of patient variability that we observed when exploring the transcriptomic profile of the edited organoids. Despite the fact that we had deleted the colorectal cancer tumour suppressor gene PTEN, the organoids clustered primarily based on the patient that they were derived from, rather than their genotype. We think that this finding is an important factor of variation and could be useful for scientists in the future when designing their experiments, as it will dictate the level of detectable transcriptomic changes between samples from different patients.

**Describe what you think is the most significant challenge impacting your research at this time and how will this be addressed over the next 10 years?**

Currently, I feel that scientists in cancer research are in a spiral of collecting large and complex multi-omic datasets, placing them in a rabbit hole of a never-ending analysis, as it can be extremely hard to analyse these data and integrate their information across all levels. It is also becoming more obvious that there is never only one way to analyse these complex datasets, and different options should be explored. I believe that, in order to respond to this challenge, a first step would be to properly equip the next generation of biomedical scientists by ensuring that bioinformatic training is a mandatory rather than an optional part of their undergraduate syllabus. Next, experiments should be designed with a focus on targeted questions rather than a general exploration of the collected datasets. Finally, a reinforcement of data-sharing opportunities and collaboration across different fields, would also be extremely valuable, opening new avenues for data analysis and interpretation.

**What changes do you think could improve the professional lives of scientists?**

We are now fortunate enough to benefit from technological advances and a plethora of datasets that allow us to address complex questions that, previously, seemed unattainable. Similarly, the standard of publications is higher, with journals expecting an increasing number of data, new methods to be described or translational perspectives to be included. Yet, the funding or contract length has not necessarily changed at the same pace. This sometimes leads to project leaders having to move on to their next role without having completed all the necessary experiments or analysis for publication. I believe that, if funders or institutes were to provide funding for a longer period of time, thereby providing better financial security for scientists (which is also a reason why a lot of people decide to eventually leave academia), time towards publication could be reduced (i.e. no need to retrain someone new) and quality of science publications could be improved (i.e. more thorough analysis and validation without the eminent time pressure).

**What's next for you?**

Having just completed my PhD, I am working on finishing my remaining publications on the somatic mosaicism of the normal human colon. Next, I will be applying for a post-doctoral position still in cancer research but perhaps focussing on a different organ, ready to work on a new challenge. After now living for almost 10 years in the UK, I am intrigued to explore a new country and make some friends in a different part of the world!
